# Unleashing the Furr-Recovery Method: Interacting with Pets in Teleworking Replenishes the Self’s Regulatory Resources: Evidence from a Daily-Diary Study

**DOI:** 10.3390/ijerph20010518

**Published:** 2022-12-28

**Authors:** Ana Junça-Silva

**Affiliations:** 1 Business ISCTE—Instituto Universitário de Lisboa, 1649-026 Lisboa, Portugal; ana_luisa_silva@iscte-iul.pt; 2 IPT—Instituto Politécnico de Tomar, 2300-313 Tomar, Portugal

**Keywords:** recovery, micro-breaks, mental health, pets, human–animal interactions, furr-recovery method

## Abstract

This study is based on the conservation of resources theory and the recovery step model in order to further explore the furr-recovery method—a mechanism through which workers break their routine by taking micro-moments to interact with their “furry co-workers,” thus relieving their fatigue and tension or other negative affective states. Based on this, we argue that this method not only serves the purpose of restoring self-regulatory resources but also ameliorates mental health. Accordingly, this study aims to analyze how daily human–animal interactions during teleworking positively influence teleworkers’ mental health, via recovering their self-regulatory resources, at the within-person level. Full-time teleworkers completed multiple online surveys for 5 consecutive workdays (*N* = 211 × 5 = 1055 daily observations). Multilevel path analysis results showed that on days on which employees had more micro-moments to interact with their “furry co-workers” during the day, they experienced a higher self-regulatory capacity and felt better while working. In sum, the findings give support for the theoretical resource perspective of interacting with pets as an effective energy management strategy while at work. This research extends the theoretical understanding of regulatory resources as a cognitive mechanism that links HAIs to employee mental health. Moreover, the findings outlined here offer practical implications by highlighting the furr-recovery method, a method that teleworkers who own pets may use as a strategy during the working day to restore resources needed to be healthier.

## 1. Introduction

The recent COVID-19 crisis imposed the adoption of telework to reduce the widespread distribution of the virus, at the same time ensuring its maintenance. Telework is a flexible work arrangement that allows workers to do their job from other locations (e.g., home) through information and communication technologies [1]. Even though the virus is more contained, it seems that the flexibility afforded by teleworking likely sustains workers’ performance as with face-to-face work [2,3].

While teleworking, pet owners get an opportunity to work near their pets, or their “furry co-workers.” Indeed, pet owners appear to be at the top of the list of those who prefer to work from home [2,3], and they often describe their pets as important and cherished family members who offer solace in times of stress [3] and company in moments of loneliness [4]. When working from home, pet owners can reduce their concerns regarding their pets being home alone for too many hours, and thus reduce their worries, anxiety, or other negative states, allowing them to better concentrate on the tasks at hand [5] and making them feel better during the day [6]. Additionally, pets (especially dogs) tended to develop strong attachments to their owners during the COVID-19 crisis, as they were together for more time due to the national confinements and mandatory teleworking [2,7]; however, now, they may experience anxiety or distress if their owners are working fully in face-to-face mode, which in turn may intensify their owners’ concerns about their dogs’ welfare [7]. Indeed, the welfare issues are not just those of the owners but of the dogs, too, who may be facing more anxiety if they are now home alone due to the return of their owners to face-to-face work. When working from home, pet owners may work near their pets, interact with them (e.g., head petting, observing the pet playing, or taking the pet for a walk), and thus feel happier and contribute to the welfare of their pets as well.

The relevance of human–animal interactions (HAIs)—the interaction between humans and pets—has been acknowledged; however, few studies have explored them in the context of telework. For instance, HAIs have been demonstrated to have positive effects on health and well-being [7,8]. For instance, a great amount of HAI research has focused on the role of animals in mitigating mental health disorders, such as reliving depression/anxiety symptoms [9]. Other studies have demonstrated that HAIs reduce stress and the feeling of loneliness, provide emotional support, improve emotional regulation and executive functioning, and are a natural booster of happiness [10,11]. Indeed, despite these pieces of evidence, only recently has this attracted organizational scholars to explore how these benefits could transpose the personal/familiar domains to the work one. This has been triggered by the call for studies of Kelemen et al. [12], who emphasized the need to understand the intersection of pets in organizational daily routines and thus transpose what is already known from other scientific areas.

HAIs may include physical (e.g., head petting), affective (e.g., observing the pet), or cognitive (e.g., perceived emotional support) interactions [5,13]. By interacting with their pets, during work, individuals break their routine and create moments of respite—a micro-break similar to that which involves interactions with co-workers. 

Micro-breaks have been explored in the recovery literature and have been demonstrated as crucial moments for the individual’s rest and recovery from daily job demands and hassles [14,15]. Indeed, individuals during their working day often experience several challenges or unexpected events that make them spend resources. When this occurs, there is a period in which they have to stop and take a break to recover those lost resources. Micro-breaks are short, informal, and voluntary breaks. They are flexible in timing, duration, and frequency because they depend on how much the individual needs them [16,17]. Some examples of micro-breaks include coffee breaks and just getting up and stretching.

For instance, Chan et al. [15] recently developed a model of recovery—the recovery step model—outlining the role of micro-breaks in diverse recovery processes, among them being self-regulatory resources [16]. Self-regulatory resources are cognitive resources that are relevant for the working day because they are related to the individual’s ability to self-control their behaviors, emotions, and impulses (e.g., focus attention on the tasks even when physically or emotionally exhausted or suppress some emotion that is not supposed to be expressed) [16,18,19]. By engaging in micro-breaks, individuals restore their capacity to fully function [17] and reach fullness [20]. Despite the relevance of micro-breaks for health outcomes [10], no study has explored HAIs as a micro-break at work (see [5] as an exception).

Relying on the recovery step model, and the furr-recovery method—the recovery process through interacting with furry co-workers, that is, pets [15]—we argued that HAIs during the day are micro-breaks that can help individuals recover their regulatory resources by making them experience relaxation and calmness, and control (i.e., contributing to a self-perceived sense of control what one wants and need to do) and distracting them from work (i.e., psychological distancing from work) [5]. Additionally, based on the conservation of resources theory [21,22], we expect that these regulatory resources’ recovery will make individuals feel better and relaxed, thereby contributing to their improved mental health. We delineate arguments to show that when regulatory resources are recovered, individuals feel resourceful, which may attenuate negative affective states, such as tension or stress, thus improving their mental health.

This study has three major contributions. First, it contributes to extending the recovery literature by unleashing the role of HAIs at work. Exploring how teleworkers’ interactions with their “furry co-workers” influence their mental health will develop a better understanding of how they may have access to unique resources while working from home—that they would not be able to have if they were working at the office. Second, HAIs—as micro-breaks—may provide the needed support for workers to preserve and develop their regulatory resources that may support how HAIs may improve mental health. Moreover, this indirect path highlights two resources that may support managers and employees in better coping with negative and uncertain conditions: telework and interacting with pets as moments to respite. Third, due to the COVID-19 pandemic crisis, many organizations across the world adopted telework as an organizational strategy; however, there are some who resist it. From a practical standpoint, it will be helpful to clarify the role of telework in workers’ mental health, in particular for those who own pets.

## 2. Theoretical Framework

### 2.1. The Importance of Pets

Pets are increasingly present in modern families. Plus, how families treat and see their pets has also changed as they have been often described as cherished family members who accompany the family in their daily routine [23]. 

These changes might be supported by the great number of empirical demonstrations of the pets’ benefits for all ages [6]. For instance, some studies have consistently demonstrated that pets help individuals to feel calm, mindful, and present in their daily life [23]. They also reduce loneliness and improve the quality of life of elderly people [24]; make kids more active, confident, and responsible [25]; and reduce the blood pressure and heart rate, raise survival chances after a heart attack, and facilitate social contact [6] and at the same time improve oxytocin levels—the known “love and attachment hormone” [26], among other benefits. In addition, pets have also benefitted an individual’s mental health as it has been demonstrated that pets reduce psychological impairment states, such as anxiety, depression [27], and psychological distress [6]. In addition, there is also plenty of evidence showing that by interacting with their pets, individuals tend to feel emotionally supported and abstracted from their problems [28].

Human–animal interactions (HAIs) are not a new research topic for psychology or medicine areas, as there is plenty of evidence of their beneficial effects on an individual’s health [29], mental health [30], well-being [31], and plenitude [13]. HAIs have been often defined as all the interactions between humans and non-human beings [28] and may include physical (e.g., going to take a walk with the pet), affective (e.g., observing the furry friend playing with a bone), or cognitive (e.g., perceived support by having the furry friend nearby) interactions [2,5,9]. 

As we outlined before, HAIs appear to have diverse benefits for individuals and include, for instance, well-being [6]. One benefit that has been highlighted consistently across studies is the health benefit of HAIs, as there are diverse studies that have shown that interacting (physically, cognitively, or affectively) with pets typically reduces depression symptoms, the stress triggered by negative events, and loneliness [28]. Moreover, HAIs, such as taking a walk with the pet, act like an “ice-breaker” as this catalyzes communication and enhances opportunities for social exchange, which in turn enhance social interaction or social skills and promote the feeling of social integration [4,10,32,33,34]. Research has also shown that the simple act of looking at the pet decreases anxiety and exerts a calming or de-arousing influence [33]. Indeed, the mere observation of a pet can attenuate physiological and psychological responses to negative and stressful situations, attenuating those stressful and anxious responses: for instance, it has been observed that the presence of a companion dog as well as interactions with friendly but unknown dogs momentary decrease the blood pressure and heart rate in individuals of different ages [8,12] (for an exhaustive review, see [9]). Moreover, other studies have shown that HAIs produce oxytocin, which impacts the central nervous system and in turn diminishes behavioral and neuroendocrine responses to distress (e.g., [35,36]). Indeed, there is increasing evidence suggesting that interacting with a pet appears to be as calming as is reading a book in silence (by lowering cortisol levels) [11,37].

### 2.2. Human–Animal Interactions as a Micro-Break during Work

As we can see, HAIs appear to be beneficial in several ways for an individual’s daily life. The impact of HAIs on the organizational domain only recently started to attract scholars who have recognized the importance of pets, not only for the personal or familiar domain, but also for the work-related one [38]. This might be related to the fact that many organizations worldwide have started to adopt organizational strategies (e.g., Amazon, Google) that include pet-friendly policies (e.g., teleworking) as a way to motivate and engage their workers in their work [2,14,38,39] and have identified diverse benefits in doing it, such as higher performance levels, increased organizational commitment and identification, and lower turnover intentions [26,40]. 

Among the different pet-friendly policies, teleworking is the most frequent one [5], in part because not all organizational spaces are prepared to receive their worker’s furry co-workers and also because workers with pets tend to prefer working from home, even in a hybrid regime—working from home some days and working at the office on other days—than working at the office in a full-time regime [2]. Indeed, pet owners tend to choose to telework instead of going to the office, because when working from home, they do not need to feel worried about their pets, who are alone at home for so many hours, which on the one hand allows them to be more focused on the tasks they have to do and on the other hand makes them feel better. Additionally, when working from home, pet owners get an opportunity to spend more time with their “furry co-workers” as they can work near them, which on the one hand makes them feel emotionally supported and, even if physically lonely, feel accompanied; on the other hand, they can interact with their pets frequently during the working day. Hence, interacting with pets during the working day means that HAIs transpose the personal domain to the working one. 

Despite the scarcity of studies exploring HAIs in the working context (for an exception, see [5]), we argue that daily HAIs are micro-breaks like those that encompass interpersonal interactions (e.g., taking a break to call to someone or to text someone) or similar to micro-breaks that include coffee breaks. Accordingly, we also assume that daily HAIs, as micro-breaks from work, serve the function of helping the individual to recover resources, such as self-regulatory resources, lost while performing the job and facing daily demands and challenges. By taking micro-breaks that involve interacting with their furry co-workers, teleworkers can regain their resources, such as energy—a resource linked to self-regulatory resources (the regulatory ability to self-control diverse behaviors, emotions, and impulses) [20]. 

Self-regulatory resources are relevant for individuals as they need them to do their jobs and deal with the diverse hassles or challenges that appear throughout the day and thereby demand a strong regulation of affect and cognition [16,41]. In addition, self-regulatory resources are limited in nature because while working, there is a natural use of such resources for different self-control tasks (e.g., stopping to procrastinate or stopping to chat, allocating and redirecting cognitive attention to the tasks) [42,43]. When this happens, individuals must engage in recovery behaviors as a strategy to stop such regulatory efforts and thus take micro-breaks to recover the depleted resources before going to the next set of self-regulation activities [16,17]. 

Micro-breaks have been explored in the literature on recovery from work. This is divided into two main streams of research: recovery after work, which includes experiences of relaxation, control, mastery, and psychological detachment from work, and recovery during work, which is mainly focused on micro-breaks aimed at recovering resources needed for the working day. 

Recently, Chan et al. [15] developed the recovery step model. Accordingly, micro-breaks are important for individuals to recover resources spent while working. Self-regulatory resources are among the most relevant recovered cognitive resources [15] and may be restored through recovery experiences (i.e., control, relatedness, mastery, enjoyment, detachment, and relaxation) [17]. Hence, micro-breaks are “short and informal breaks/respite activities taken voluntarily between tasks” [16], (p. 773) that are generally considered more flexible in timing, duration, and frequency and are typically self-initiated [15]. In addition, such pauses are a way to stop resource spending and renew other ones [15], serving as a resource-replenishing strategy that is relevant between different task episodes [16,17]. Micro-breaks are relevant because they may include experiences that (1) bring relaxation and calmness to the individual (e.g., calling a friend in search of emotional support), (2) improve the sensation of control (e.g., taking a coffee break), (3) create psychological detachment from some task or problem at work (e.g., taking a walk, interacting with colleagues about off-job activities), or (4) create a sense of mastery (e.g., through pleasurable micro-activities, such as looking to improve knowledge through reading).

Empirically, some studies have shown that micro-breaks decrease tiredness and improve resources needed for daily activities (see [18,44]). This happens because while working, individuals exert efforts, which leads to a loss of resources [45], either by performing their tasks or by having to make efforts to deal with daily work demands. This is why they need to take some breaks during the working day—to recover what is lost. This is supported by the conservation of resources theory [46]. Accordingly, individuals when perceiving a loss of resources engage in behaviors to recover them—for instance, taking a micro-break from work—as they are impelled to maintain, acquire, or develop resources [46,47]. For them, resources are needed to face daily challenges that may be stressful or harmful to their mental health. Hence, the act of preserving or searching for resources is an ongoing daily behavior whose objective is to avoid entering the spiral of resource losses. These acts are often relied on when taking micro-breaks that help them to enlarge and preserve the resource reservoir. When micro-breaks are positive and help to recover lost resources, individuals turn into a state of resourcefulness that improves their ability to focus on what they have to accomplish and makes them feel better. 

### 2.3. The Mediating Role of Regulatory Resources

Diverse micro-breaks have been explored in the literature, (e.g., coffee or tea breaks, micro-interactions with co-workers) [15,16]; however, HAIs in the work context have been less studied. Relying on the step recovery model, we argue that HAIs may help teleworkers to recover self-regulatory resources, and in turn, this may promote their mental health. 

First, physical HAIs, such as touching or petting the head of a furry friend, are physiologically and emotionally pleasurable for the individual. With respect to this, Olmert [48] suggested that the urge to touch an animal is biological, and this occurs even for unknown pets. For instance, neuroscience studies have shown that simply looking at a dog or stroking or talking to a dog can release oxytocin. It has been consistently demonstrated in the literature that oxytocin decreases the production of stress hormones and diminishes the experiences of fear and danger [11,12,48]. As such, oxytocin not only is responsible for the individual to feel pleasure but also helps them to broaden and restore other resources, such as cognitive resources needed to self-regulate actions, emotions, or impulses.

Second, another stream of research has evidenced that a simple eye exchange between humans and pets leaves them with the feeling of being supported and makes them experience positive emotions, such as calm and relaxation [23]. There is theoretical and empirical evidence for the influences of positive emotions on an individual’s behaviors [47]. Accordingly, positive emotions are personal resources with their own value; they serve to broaden an individual’s cognitive and behavioral repertoire, which, in turn, improves their ability to acquire and develop other resources that are enduring in nature [47]. 

Third, as mentioned earlier, although some pets with behavioral issues may be a nuisance to their owners, ongoing pet ownership generally suggest that pets are not only cherished family members but also unique resources due to their attachment role. This has been demonstrated in interviews with pet owners who described their relationship with their pets as caring and nurturing and to whom they are emotionally tied [6]. Theoretically, the attachment experiences give support to these studies [49]. Accordingly, emotional bonds are processed and stored in the right hemisphere of the brain, influencing affective (e.g., mental health) and cognitive (e.g., self-regulation) functioning [49]. Such benefits have been described in the popular media, particularly in the recent event of the death of Queen Elizabeth II. Although this is not a scientific example, it is relevant to consider. For instance, close friends and familiars of the queen reported that in stressful and tense moments, her refuge was in her furry friends—the corgis—as she saw in them a unique way to relieve her anguish. Her family called it the dog mechanism: “[…] If the situation becomes too difficult, she will sometimes literally walk away from it and take the dogs out” [50]. 

**Figure 1:**
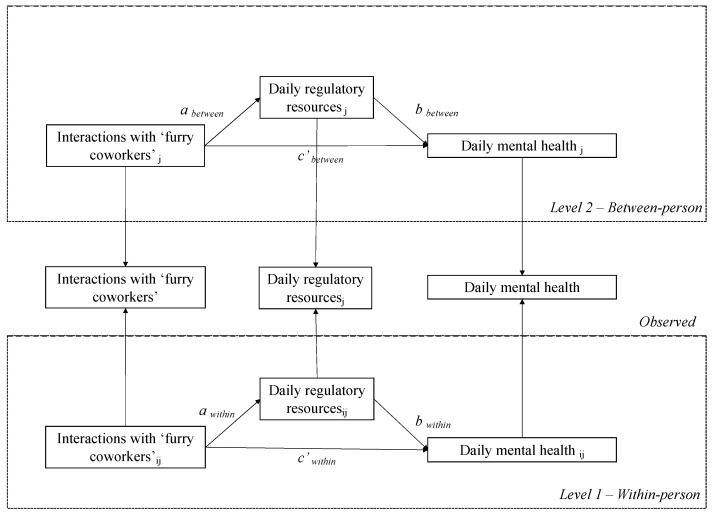
The conceptual model under study. For brevity, the control variables are not shown in the framework.

Hence, relying on the recovery step model, we argue that interacting with their “furry co-workers” may help individuals to replenish their self-regulatory resources, which will contribute to improving teleworkers’ mental health [51]. Based on the conservation of resources theory (COR), we hypothesized that for the within-person level, individuals tend to have more self-regulatory resources on days in which they engage in more interactions with their furry co-workers (H1a) and that this will serve as a mechanism explaining why HAIs enhance their mental health (H2a). Moreover, at the between-person level, we expect that individuals with higher average levels of HAIs tend to have more self-regulatory resources than individuals with lower average levels of daily HAIs (H1b). In addition, these average levels of self-regulatory resources will serve as a potential mechanism for the relationship between daily HAIs and daily mental health at the between-person level (H2b); see Figure 1.

**Hypothesis** **1** **(H1).** *Daily HAIS will be positively related to daily regulatory resources at the (a) within- and (b) between-person level.*

**Hypothesis** **2** **(H2).** *The relationship between daily HAIs and daily mental health will be mediated by daily regulatory resources at the (a) within- and (b) between-person level.*

## 3. Methods

### 3.1. Participants and Procedure

In total, 211 individuals who were teleworking participated in the study. They included human resources managers (37%), advertisers (33%), trainers (22%), and researchers (8%). Overall, 64% were female, the mean age was 38.50 years (SD = 10.32), and the mean tenure was 16 years (SD = 6.78). On average, they worked about 41 h per week (SD = 6.13). All participants had pets (M = 3.2, SD = 3.70) living with them. Dogs were the most reported pets (92%), followed by cats (21%). Overall, 28% had both dogs and cats. On average, the teleworkers reported having pets at 16 years (SD = 14.11).

The researcher asked the teleworkers from their professional network to participate in a study about telework attitudes. The ones who agreed to participate were explained the main goals and the data collection procedure. Moreover, in a second email, they were assured that their participation was completely voluntary and anonymous and that their responses would be confidential. Next, they signed an informed consent form before answering the general survey. After this, they received the hyperlink for the general survey, which assessed the participants’ sociodemographics and their pets’ characteristics. In the following week, they started the daily questionnaires (collected once per day at the end of the working day) for 5 consecutive days (from Monday to Friday). Each participant received a daily email at 6:00 p.m. with the hyperlink for the daily survey. They had to answer it by 10:00 p.m. On average, they answered it at 7:30 p.m. Of the 255 teleworkers who agreed to participate, 211 provided valid responses across the 5 days (*n* = 1055; response rate = 83%). 

### 3.2. Measures

#### 3.2.1. Human–Animal Interactions 

Human–animal interactions were measured with four items developed by Junça-Silva et al. [2]. An item example is “Today while teleworking I took breaks to interact with my pet.” Participants used a 5-point scale (1 = never; 5 = four times or more). Multilevel reliability performed through the Alpha and the Omega index suggested that the high values (α_between_ = 0.93, ω_between_ = 0.93; α_within_ = 0.96, ω_within_ = 0.96) may potentially suggest that some items are measuring the same thing.

#### 3.2.2. Daily Self-Regulatory Resources

To assess daily self-regulatory resources, we used the 3-item Regulatory Resource Availability scale [52] (e.g., “Today, I have not been feeling mentally energetic.”). Responses were given on a 5-point Likert scale ranging from 1 for never to 5 for always. Multilevel reliability tests indicated acceptable reliability (α_between_ = 0.84, ω_between_ = 0.85; α_within_ = 0.86, ω_within_ = 0.86).

#### 3.2.3. Mental Health

To measure the participants’ daily mental health, we used three items from the SF-36v2 Health Survey [53]: “Today, how much of the time have you felt calm and peaceful?” Items were rated on a 5-point scale ranging from 1 (none of the time) to 5 (all of the time). Multilevel reliability indices were good (α_between_ = 0.70, ω_between_ = 0.71; α_within_ = 0.66, ω_within_ = 0.63).

#### 3.2.4. Control Variables

The time of data collection (from Monday to Friday) was a daily-level control variable once it was found that while the study was ongoing, there was an influence on the criterion variables, known as the learning effect [54]. Sex and the number of pets were between-person-level control variables because the number of pets may influence daily HAIs and subsequent regulatory resources (as it may lead to a higher number of volatile actions to interact with them) and sex may influence both regulatory resources and performance-related outcomes.

### 3.3. Data Analysis

This study used multi-level analysis with nested data to examine the underlying model. We found significant variance in daily HAIs (ICC = 0.52), regulatory resources (ICC = 0.56), and mental health (ICC = 0.65). This evidenced that these variables have significant variation at both within- and between-person levels. Thus, we proceeded with the multilevel analysis.

Before testing the hypotheses, we analyzed the issue of the common method variance in this study, because even though this was a daily-diary study, the predictor, mediator, and criterion variables were measured at the same time. First, we need to highlight that throughout the daily survey, we shuffled the questions of various measures and then used various dummy questions (e.g., I like horror movies). Second, we tested the factorial structure of the data through multilevel CFAs using Jasp software, version 0.16.4. We first tested a three-factor model with the three multi-item variables under study (HAIs, self-regulatory resources, and mental health). The three-factor solution yielded a good fit (χ^2^ = 116.73; *p* < 0.001; df = 60; RMSEA = 0.06; CFI = 0.99; TLI = 0.99; SRMR_within_ = 0.05; SRMR_between_ = 0.05). The model fitted better than a two-factor model (where HAIs and self-regulatory resources loaded on one factor; χ^2^ = 1654.62; *p* < 0.001; df = 62; RMSEA = 0.24 CFI = 0.98; TLI = 0.97; SRMR_within_ = 0.23; SRMR_between_ = 0.24) and a one-factor model (where all items were loaded on a single factor; χ^2^ = 2495.39; *p* < 0.001; df = 63; RMSEA = 0.29; CFI = 0.97; TLI = 0.96; SRMR_within_ = 0.27; SRMR_between_ = 0.27). Thus, the current three-factor structure was valid. These results and the reliability scores evidenced the discriminant and convergent validity of the study; hence, we proceeded with the test of hypotheses.

The hypotheses were tested through the macro–Multilevel Mediation (MLMed) in SPSS [55]. This is a suitable macro to test the hypothesized 1-1-1 multilevel mediation model (daily HAIs → daily self-regulatory resources → daily mental health) because it appears to deliver similar results, in the estimation of the model’s parameters, to what other software alternatives do (e.g., Mplus). These confidence intervals are significant when they do not include zero. 

## 4. Results

### 4.1. Descriptive Statistics

Table 1 shows the descriptive statistics and correlations between the variables under study.

### 4.2. Hypothesis Testing

As we mentioned before, to test our hypotheses, we considered the hierarchical structure of the data, in which daily data were nested within individuals. 

Hypothesis 1 expected that daily HAIs would positively influence daily self-regulatory resources at the within- and between-person levels. Daily HAIs were positively correlated with daily self-regulatory resources (γ = 0.11, *p* < 0.01). However, the between-person results showed that daily HAIs negatively affect daily regulatory resources (γ = −0.15, *p* < 0.01). Thus, the first hypothesis was only partially supported, as the between-person hypothesis was supported but in the opposite direction. 

Next, we moved to hypothesis 2. This hypothesis assumed that daily HAIs would positively influence daily mental health through daily regulatory resources at both within- and between-person levels. Even though daily HAIs did not present a significant direct relationship with daily mental health, we proceeded with the mediation analyses because there are authors who argue that even in situations where the independent variable is not significantly related to the dependent one, there may be a mediation [56]. For instance, Rucker et al. [56] argued that rather than analyzing only the paths between variables, attention should be shifted toward assessing the magnitude and significance of indict effects. Hence, we proceeded with the analysis of the indirect effect.

**Figure 2:**
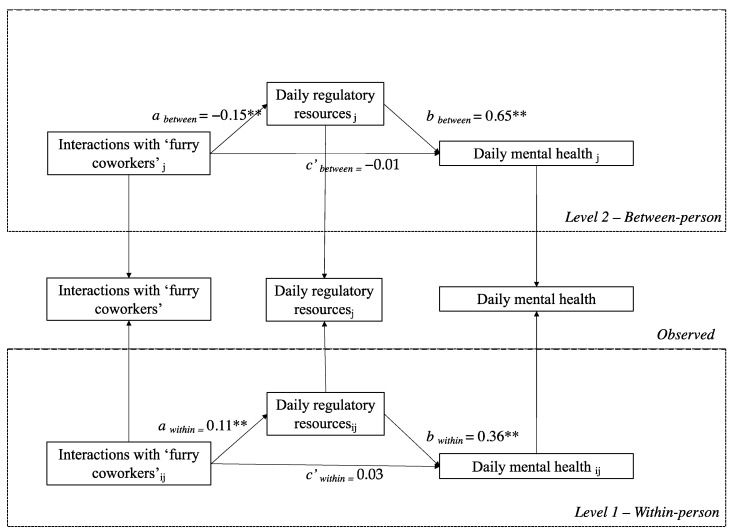
The multilevel mediating model. Maximum-likelihood estimation with robust standard errors (MLR) was used in the estimation. L_1_ = level 1 (1055), L_2_ = level 2 analysis (211). ** *p* < 0.01. *R*^2^ = 25.7. AIC = 4705.11; BIC = 4735.47; −2LL = 4705.11.

The multilevel results showed a significant indirect effect of daily regulatory resources at both between- and within-person levels (Estimate_between_ = −0.10, *p* < 0.01, 95% CI [−0.18, −0.02]; Estimate_within_ = 0.02, *p* < 0.01, 95% CI [0.01, 0.07]). Thus, (H2) was supported by the data; however, the between-person indirect effect occurred in the opposite direction to what was expected (see Figure 2).

## 5. Discussion

Theoretical models have suggested that the recovery process is of crucial importance for individuals’ respite from work-related challenges and hassles. Moreover, these models highlight the relevance of taking micro-breaks along the day to recover resources. However, current models do not identify micro-breaks that encompass human–animal interactions. Empirically, the benefits of HAIs for an individual’s health are consistently demonstrated (e.g., [6]); however, organizational scholars have only recently started to focus on the intersection of pets and daily life at work (e.g., [5,14]). As such, the inclusion of HAIs as micro-breaks in teleworking settings has not received attention. This paper addresses these issues and contributes to a better understanding of HAIs, their role as micro-breaks that restore self-regulatory resources, and how they may impact teleworkers’ mental health by conceptualizing and testing this process on both levels (within- and between-person levels).

This study relies on the step recovery model by conceiving daily HAIs during telework as informal micro-breaks that create moments for workers to rest. Additionally, the study is based on the conservation-of-resources perspective by outlining the nature of daily HAIs as a strategy to preserve and acquire self-regulatory resources that are needed for individuals to feel mentally healthy. Hence, this study extends previous research on HAIs to demonstrate that (1) they serve the function of helping an individual to recover self-regulatory resources and (2) in turn they contribute to better indices of mental health. 

### 5.1. Theoretical Implications on HAIs and Recovery from Work

The recovery process from work has received attention from scholars who have empirically demonstrated that a working day is filled with challenges or daily hassles [56] that gradually spend workers’ resources, leading them to feel mentally fatigued or exhausted [57]. Moreover, these studies have also evidenced that the process of engaging in behaviors to stop working—micro-breaks—allows the individual to recover what has been lost while performing the job [15]. 

Even though having a pet is bright and shining, those who have them might also experience some challenges by being involved in caring for them while working from home (e.g., the cat sitting on their lap) or some pet-related hassles (e.g., dogs barking during meetings, having to go outside at an inconvenient time). Notwithstanding, those who have pets stress that these are minor issues when compared to the good things they provide.

Indeed, those who work from home and own pets may engage in micro-breaks by simply looking at them or interacting with them. This study extends previous research by demonstrating that micro-breaks may also include HAIs in telework settings. The findings support the within-person-level results, but the between-person-level findings are contradictory to what was expected. That is, teleworkers tend to have more self-regulatory resources on days in which they engage in more interactions with their pets, and this explains why they feel mentally healthy on those days. This means that while working from home, individuals may create informal moments that serve as respite breaks. These breaks, which involve physical or affective interactions with their furry co-workers, help individuals recover their self-regulatory resources—a limited resource that is required for self-control (e.g., stop snacking)—and this leads to improved mental health on a daily basis. The benefits of HAIs for health are commonly known (e.g., [6,28]); however, there is a lack of demonstration in the organizational literature. In addition, teleworking appears to be an important setting that privileges these “furry” interactions that appear to be resourceful and protective of mental health. Thus, these findings are relevant because not only expand the step recovery model [15] by including HAIs as micro-breaks but also contribute to demonstrating how these moments can deliver health benefits for teleworkers. Moreover, by demonstrating that, we also contribute to expanding the HAI research to the organizational context. 

However, the findings from the between-person level suggest that on average, teleworkers who engage in more interactions with their pets tend to lose self-regulatory resources, but this appears to protect their mental health. In other words, teleworkers who report fewer HAIs tend to have more self-regulatory resources; however, their mental health is, on average, worse. Thus, engaging in HAIs may lead to a decrease in self-regulatory resources but mental health is protected. This might occur because in fact stopping work to engage in HAIs may imply some self-regulatory resources to do it, which means that one needs to spend self-regulatory resources to interact with the furry co-worker. However, even with spending self-regulatory resources, mental health improves. Thus, spending self-regulatory resources—or investing cognitive, emotional, and behavioral resources to achieve a desired goal or outcome—is not always a bad thing, because it may indeed be necessary to protect teleworkers’ mental health. As Baumeister and Heatherton (1996) emphasized, when individuals self-regulate, they attain some gratification and pleasure, which may explain the self-regulatory decreases but the resultant increases in mental health. Hence, the hedonic approach of searching for some gratification in HAIs appears to be favorable in the long run; despite the loss of regulatory resources in the short run, it leads to improved mental health in the long run. 

In sum, this daily-diary study demonstrates that pets may indeed be “furry co-workers” because similarly to the interactions that individuals have with human co-workers, HAIs appear to have benefits at both within- and between-person levels. In addition, HAIs appear to be informal moments of respite, or the so-called micro-breaks that serve to recover self-regulatory resources. Even though in the between-person analysis, there appears to be some self-regulatory resource loss, in the long run, these losses are beneficial for the individual. Moreover, it is important to emphasize the role that HAIs, as micro-breaks, play both in the self-regulatory reservoir and in the individual’s mental health. Hence, this study further underscores the furr-recovery method by highlighting the positive impact that HAIs have on resource recovery and transposing it for the worker’s mental health. 

### 5.2. Practical Implications

The results of this study are relevant for managers who wish to improve their workers’ mental health. First, teleworking as a flexible working arrangement [58] appears to be a unique context in which workers who own pets, and who do not have organizations physically prepared to take them, may benefit from their presence. Hence, managers may thereby analyze which workers own pets and decide accordingly. Moreover, teleworking may not only promote unique conditions for HAIs but, as a result, also appear to be a resourceful and mentally healthier context.

Additionally, the furr-recovery method was demonstrated to be beneficial for performance [5] and mental health; hence, it appears to be an opportunity for organizations that intend to adopt pet-friendly policies, such as teleworking, or implement a “pet day at work.” By doing so, organizations may improve their results regarding performance and contribute to being a healthier place to work. In addition, this may improve their image and contribute to their positive employer branding and, in turn, increase retention rates and decrease turnover.

### 5.3. Limitations and Future Research

Several factors increase our confidence in our results. First, this study was a daily diary that analyzed within- and between-person effects. This analysis is important because there may be differences between each kind of analysis. In addition, as we outlined earlier, there are slight differences between within- and between-person levels. Moreover, this study had a good sample size, which is relevant for the generalizability of the findings. 

Despite these strengths, some limitations should be mentioned. First, the self-reported nature of the data was a major limitation as it may lead to common method bias [59]. Even though we took some measures to prevent it, it may be a source of limitation associated with the findings. Moreover, it is relevant to emphasize that self-report measures are a reliable way to assess inner states as self-regulatory and mental health [60,61,62]. Second, all the variables were measured at the same time of the day—at the end of the working day—which may limit the generalizability of the results. As such, future studies should rely on daily-diary studies with multiple time points, for instance, collecting data at lunchtime and at the end of the working day. 

This study opens avenues for further research. It should be relevant to explore whether the furr-recovery method occurs in the office settings for organizations that allow their workers to take their pets to work. Moreover, researchers should also focus on exploring when the furr-recovery method tends to be intensified by analyzing personality characteristics that may moderate the relationship between HAIs, self-regulatory resources, and resultant outcomes (e.g., performance or mental health).

## 6. Conclusions

This study underscores the furr-recovery method by showing that HAIs are resourceful and help to improve teleworkers’ mental health. Together, the findings suggest a more complex picture of the health-related effects of HAIs. Whereas daily related fluctuations in HAIs seem to exert positive effects on self-regulatory resources and mental health, HAIs on an aggregated level seem to dampen self-regulatory resources but at the same time contribute to improving mental health, thus suggesting beneficial effects for health.

## Figures and Tables

**Table 1:** Means, standard deviations, and between- and within-person-level correlations.

Variables	M	SD	1	2	3	4	5	6
1. HAIs	1.61	1.00	-	0.06 *	0.05	0.06 *	0.22 ***	0.10 **
2. Regulatory resources	3.70	0.95	0.10 *	-	0.51 ***	0.03	0.00	−0.06 *
3. Mental health	3.55	0.92	0.01	0.56 ***	-	−0.00	0.05	0.00
4. Time	-	-	0.05	−0.01	0.06 *	-	0.05	0.02
5. Number of pets	3.20	3.70	0.32 **	0.07 *	0.00	0.04	-	0.05
6. Sex	-	-	0.14 *	0.04	−0.07 *	0.01	0.05	-

Note. Correlations below the diagonal are between-person levels. Correlations above the diagonal are repeated-measures correlations (rmcorr). Sex: 1 = male; 2 = female. *N*_(observations)_ = 1055; *n*_(participants)_ = 211. *** *p* < 0.001, ** *p* < 0.01, * *p* < 0.05.

## Data Availability

Data will be made available upon reasonable request.
